# Gestational Hypertension and Human Breast Milk Composition in Correlation with the Assessment of Fetal Growth—A Pilot Study

**DOI:** 10.3390/nu15102404

**Published:** 2023-05-21

**Authors:** Ewa Magdalena Sokołowska, Joanna Maria Jassem-Bobowicz, Izabela Drążkowska, Zuzanna Świąder, Iwona Domżalska-Popadiuk

**Affiliations:** 1 Scientific Students’ Circle, Division of Neonatology, Medical University of Gdańsk, 80-210 Gdańsk, Poland; zuzanna.swiader@gumed.edu.pl; 2 Division of Neonatology, Medical University of Gdańsk, 80-210 Gdańsk, Poland; izabela.zapasnik@gumed.edu.pl (I.D.); idomzal@gumed.edu.pl (I.D.-P.)

**Keywords:** gestational hypertension, human milk, lactation, breast milk composition, macronutrients

## Abstract

Background and aims: 1 in 10 pregnant women is diagnosed with gestational hypertension. Increasing evidence suggests that preeclampsia, gestational diabetes and gestational hypertension may affect human breast milk’s lactogenesis and percentage composition. We aimed to examine whether there is any significant influence of gestational hypertension on the composition of macronutrients in human breast milk and to assess its correlation with fetal growth. Methods: A total of 72 breastfeeding women (34 diagnosed with gestational hypertension and 38 normotensive women during pregnancy) were recruited to the study at the Division of Neonatology, Medical University of Gdańsk, between June and December 2022. Milk samples were collected between the 3rd and 6th day of lactogenesis. Samples were analyzed using the Miris HMA™ Human Milk Analyzer (Upsala, Sweden), which evaluates the milk composition’s energy, fat, carbohydrate and protein quantity. In addition, we assessed the children’s anthropometric measurements (birth weight, body length and head circumference at birth). We used logistic regression to estimate the adjusted odds ratio and 95% confidence interval. Results: The mean (±standard deviation) macronutrient composition per 10 mL of milk in the GH group was 2.5 g (±0.9) of fat, 1.7 g (±0.3) of true protein, 7.7 g (±0.3) of carbohydrates and 63.2 g (±8.1) of energy, in the normotensive women group 1.0 g (±0.9) of fat, 1.7 g (±0.3) of true protein, 7.3 g (±0.4) of carbohydrates and 57.9 g (±8.6) of energy content, respectively. The fat composition was higher in the PIH group by a mean of 0.6 g (*p* < 0.005). Gestational hypertension had a positive, significant correlation with birth weight (*p* < 0.013) and the mother’s pre-pregnancy weight (*p* < 0.005). Conclusions: In conclusion, we found significant differences between milk composition in postpartum women with gestational hypertension compared to healthy, normotensive women. Human milk from women with gestational hypertension was found to contain a higher composition of fat, carbohydrates and energy in comparison to healthy women. Our aim is to further evaluate this correlation, as well as to assess the growth rate of newborns in order to determine the need for individualized formulas for women with pregnancy-induced hypertension, those with poor lactogenesis and those who cannot or choose not to breastfeed.

## 1. Introduction

Gestational hypertension (GH) has been estimated to affect approximately 18 million pregnancies worldwide [1]. GH, along with proteinuria, results in the development of preeclampsia, a severe multisystem disorder occurring during pregnancy. Preeclampsia affects 2 to 10% of pregnant women [2] and is one of the primordial causes of maternal, fetal and neonatal mortality and morbidity [3,4]. Children from pregnancies complicated by GH are at greater risk of growth retardation and metabolic and endocrine disorders, and are more likely to develop neurological deficits such as cognitive impairments as a result of vascular flow disturbance at the stage of intrauterine development [5]. Studies show that newborns from pregnancies with induced hypertension are, most often, born preterm, resulting in lower birth weight as well as more severe complications associated with prematurity [6,7].

The body of a pregnant mother undergoes multiple changes to meet the needs of a growing fetus. The pathogenesis of gestational hypertension has not yet been fully explored. Nevertheless, recent studies have evaluated its possible impact on the secretory function of the mammary glands, thus affecting the composition of human breast milk, the primary source of nutrition for a neonate. Human milk (HM) contains a range of macronutrients and metabolites which are essential for the proper development of an infant [8,9]. These elements are either supplied externally, from the diet, or synthesized in the mammary glands [9,10]. Studies show that development of mammary glands is influenced by angiogenesis, local hormones and certain growth factors which may be altered in women with gestational hypertension [11]. Furthermore, impaired vascularization of the fetoplacental unit may increase the synthesis of maternal triglyceride levels, resulting in triglyceride-rich residual lipoprotein accumulation and platelet activation. This leads to endothelial dysfunction, peripheral vasoconstriction and decreased arterial compliance, which eventually manifests as hypertension [12,13]. Recent studies have also shown correlations with abnormal lipid metabolism within woman with preeclampsia. This occurs via a reduction in the catabolism of triglyceride-rich lipoproteins, mainly due to reduced placental uptake [14]. Consequently, high pressure may affect the development of the mammary gland, influencing lactogenesis and, as a result, bringing about changes to the macronutrient composition of human breast milk.

HM is natural source of nutrition, and is considered to be the most beneficial for the proper growth and development of a newborn [15]. Breast milk is the richest source of easily digestible macronutrients and hormones critical for the proper growth and development of an infant. Lipids are primarily used for the maturation of the central nervous system, both in the formation of cell structures as well as myelin fibers. Endogenously, the mammary gland produces long-chain polyunsaturated fatty acids (LC-PUFA) needed to form neuronal cell membranes. Fats, in addition to their primary function, which is providing energy to newborns, are also a source of essential fatty acids and allow for the absorption of fat-soluble vitamins. The second group of important macroelements is carbohydrates. Glucose is distinguished as the most important and only physiological energy substrate for brain cells. Exclusive breastfeeding is recommended in the first 6 months postpartum, with evidence supporting its continuation for up to 2 years or longer [16].

There are limited studies focused on assessing the impact of GH on macronutrient quantity in HM. Recently, there have been more advanced studies showing the differences in specific milk metabolites using NMR spectroscopy [17]; yet, this technique is not easily available and requires more advanced laboratory techniques. We, therefore, conducted a study among postpartum women with gestational hypertension to (1) evaluate associations between gestational hypertension and fetal growth rate and (2) search for any significant differences in the milk macronutrient composition of women with gestational hypertension using the Miris HMA™ Human Milk Analyzer (Upsala, Sweden).

## 2. Materials and Methods

This project is a prospective, single-center, hospital-based cohort study. The data presented in this manuscript represent the preliminary analysis of the first group recruited and analyzed in the study. Breast milk samples were collected from 34 mothers with gestational hypertension and 38 normotensive women. The subjects of the present study were admitted to the Department of Obstetrics and Gynecology of the tertiary perinatal unit between June and December 2022. Thus far, 34 patients had been recruited as the study group. We included postpartum women at least 18 years old between the 3rd and 6th day of lactation who had previously been diagnosed with gestational hypertension based upon the criteria from the Guidelines on Diagnoses and Treatments of Hypertensive Disorders in Pregnancy by European Society of Cardiology, ESC [18,19]. Gestational hypertension is defined as the occurrence of systolic BP ≥ 140 mmHg and/or diastolic BP ≥ 90 mmHg, developing at or after the 20th week of pregnancy among previously normotensive women [17]. Consequently, a total of 34 women with GH were evaluated as the study cohort for the investigation: 25 mothers with mild hypertension (140–149 mmHg) and 9 women with moderate hypertension (150–159 mmHg). The patients were also asked to fill in a questionnaire including their clinical data. We excluded women with diabetes, hepatic or kidney failure, chronic hypertension, multiple pregnancies, eclampsia and those with high blood pressure occurring before the 20th week of pregnancy. The local ethics committee approved this study (Ethics Committee no: NKBBN/316/2022), and the consent of the participants were obtained prior to the collection of the samples. The research project was co-financed by the European Union from the European Social Fund under the Operational Program Knowledge Education Development, 2014–2020.

### 2.1. Collection of Samples

Ten to twenty milliliters of human breast milk were obtained from the participants and immediately stored frozen at −20 °C until further analysis. Milk samples were collected between the 3rd and 6th day of lactation, depending on the mothers’ lactogenesis. Before sample collection, the participants were obliged to sign a written consent form to participate in the project. The mothers were then given oral instructions on the standardized collection of the milk sample. The human breast milk samples were collected using an electric breast pump into a sterile plastic tube. Prior to milk collection, the nipples and mammary gland areas were cleaned with soap and water. Milk samples were collected at the same time of the day from each participant, between 12 p.m. (midday) and 6 p.m. The Miris HMA™ Human Milk Analyzer was used to determine the energy, fat, carbohydrate and protein contents in human breast milk. Before analysis, the samples were prepared by increasing their temperature in a 40 °C heater and homogenizing them using cavitation phenomenon, before injecting them into the Miris Human Milk Analyzer. Subsequently, an analysis of the macronutrient composition was performed. All of the measurements were repeated 3 times for each probe, and we used the mean values to evaluate the results in order to avoid bias.

### 2.2. Laboratory Methods

The HMA uses medium-infrared spectroscopy to sense the levels of macronutrients based on their respective wavelengths [20]. The analyzer provided a calculation of energy using the conversion factors of 4.4, 9.25 and 4.0 kcal per 100 mL for total protein, fat and carbohydrates, respectively. The total protein value refers to nitrogen × 6.25, and true protein is calculated as the total protein minus 24% for nonprotein nitrogen. Total protein was converted to bioavailable protein (true protein) for the data analysis using the following equation: total protein (grams) × 0.825. The manufacturer’s protocol was followed according to the user manual, including regular calibration, cleaning after every ten samples and checking of the machine [21].

### 2.3. Statistical Methods

Data were collected and analyzed using Microsoft Office Excel 2023 and JASP (version 0.16.4, 2018 the JASP team, Amsterdam, The Netherlands) software. All types of the t-test were used to compare the macronutrient composition in human milk between the study group and the control group. The results were expressed as averages, medians and standard deviations in the tables, and as 95% confidence intervals. Student t-test was applied, and *p*-values were calculated. Categorical and quantitative variables are given as numbers and medians (25th to 75th percentiles). A correlation analysis was performed to further examine the impact of gestational hypertension on the macroelement composition of human milk. We used logistic regression to evaluate the model for factors which were associated with the occurrence of gestational hypertension. A *p*-value < 0.05 indicated a significant finding.

## 3. Results

**Figure 1:**
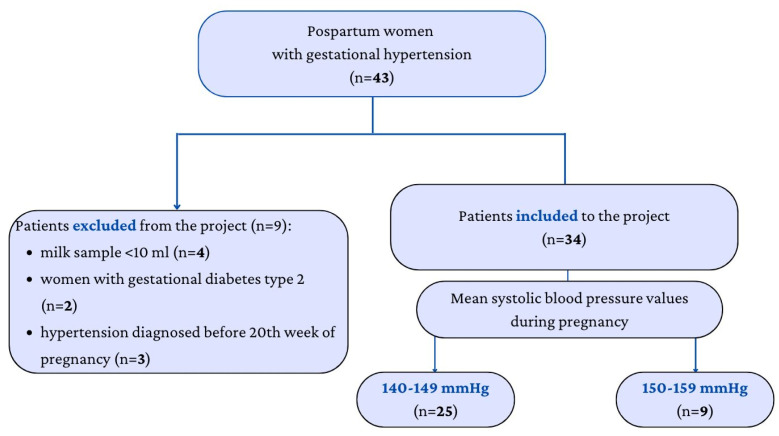
Baseline of characteristics of the study population.

Human milk samples and clinical information were collected from 34 mothers with gestational hypertension and 38 normotensive women. The patients’ characteristics are presented in Table 1 and the baseline characteristics of the study population is presented in Figure 1.

The mean age of mothers with gestational hypertension was 31 years, with a distribution median of 31 years and standard deviation of 4 years, almost concordant to the mean value for the control cohort (Table 1). Most of the respondents lived in cities of >400,000 inhabitants (48.4%), followed by women living in the countryside (21.5%), in cities between 100 to 400 inhabitants (15.1%) and in areas with less than 100 inhabitants (15.1%).

Of the respondents, 55.4% of women gave birth naturally and 44.6% underwent cesarean sections. Within those, 32.6% had an emergency C-section due to maternal or fetal complications. Gestational age (GA) was comparable in both the study and control groups: 271 days (39 weeks and 1 day) and 279 days (39 weeks and 6 days), respectively. There were two preterm pregnancies within the study group.

We noted a strong correlation between pre-pregnancy BMI and gestational hypertension (*p* < 0.005); 14.7% of the women with GH were classified as overweight (BMI 25.0–29.9 kg/m^2^) and 26.5% as obese (BMI > 30 kg/m^2^). In the questionnaire, the participants from the study group were asked to fill in their mean systolic blood pressure (SBP) values recorded during pregnancy; 25 participants (73.5%) had a mean SBP in the range of 140–149 mmHg (mild hypertension), and 9 (26.5%) between 150–159 mmHg (moderate hypertension). There was no correlation between the mean blood pressure values and HM composition. Logistic regression analysis of maternal pre-pregnancy weight status and the macronutrient composition in the colostrum of GH women demonstrated no association between the given variables. None of the *p*-values for the analyzed macronutrients was statistically significant (Table 2 and Table 3).

### 3.1. Human Milk Content

We investigated the influence of gestational hypertension on macronutrient’s composition in human milk. We analyzed the samples by their content (fat, carbohydrates, calories, true protein) with the results being given in Table 4. 

#### 3.1.1. Protein

We aimed to verify whether the concentration of protein differed between the study group and the control group. In order to check for the distribution of the variables, the Shapiro–Wilk test was used. The protein concentration variance between the two groups was not statistically significant, as show in Table 5. We also verified the relationship between the protein and fat concentration. Normotensive women had significantly higher protein-to-lipid indices than mothers with gestational hypertension (*p* < 0.003).

#### 3.1.2. Carbohydrates

We aimed to verify whether the concentration of carbohydrates differed between the study group and the control group. In order to check for the distribution of the variables, the Shapiro–Wilk test was used. We obtained significant data, as shown in Table 6. The *t*-test was performed to verify the significance of the carbohydrate content in the breast milk of women with gestational hypertension, and it showed statistical significance (*p* < 0.001).

#### 3.1.3. Fat

We aimed to verify whether the concentration of fat differed between the study group and the control group. In order to check for the distribution of the variables, the Shapiro–Wilk test was used. We obtained a significant result, as shown in Table 7. The *t*-test was performed to verify the significance of the fat content in the breast milk of women with gestational hypertension, and it showed statistical significance (*p* = 0.005).

#### 3.1.4. Energy

The composition of the energy content in human breast milk was analyzed based on samples collected from pump aspiration between the 3rd and 6th day of lactation. Based on the statistical analyses which were performed, it can be observed that the milk energy content of mothers with gestational hypertension is greater in comparison to the control group (Table 1). We performed a t-test to verify the significance of this variable (*p* = 0.005), as shown in Table 8.

We used a multinomial logistic regression model to show correlations between variables such as low birth weight, high pregnancy BMI calculated before the due date and greater carbohydrate content in the foremilk of women with gestational hypertension. The results are presented in Table 9. The results are positively and significantly correlated. A *p*-value of <0.01 indicated a significant value, according to Bonferroni correction.

## 4. Discussion

To the best of our knowledge, this is the first pilot study focusing on the association between gestational hypertension and its influence on the macronutrient composition of human breast milk (HM). During pregnancy, the body of a pregnant woman adapts, including certain changes in the nutritional and hormonal environment, to meet the requirements for proper fetal development. Gestational hypertension leads to further modifications so as to avoid fetal and maternal complications. We have demonstrated a positive correlation between the occurrence of gestational hypertension and increased fat, carbohydrate and energy content in HM: specifically, our results showed 2.5 g (±0.9) of fat, 7.7 g (±0.3) of carbohydrates and 63.2 g (±8.1) of energy content versus 1.0 g (±0.9) of fat, 7.3 g (±0.4) of carbohydrates and 57.9 g (±8.6) of energy content in the breast milk of normotensive women, respectively. However, expression patterns and maternal factors have a significant impact on the composition of human breast milk. The human milk content is not only dependent upon the individual maternal characteristics, but also varies depending on the time of feeding and the overall course of lactation. Studies show that fat is the most variable HM nutrient. In our study, milk samples were collected in the afternoon (12 pm to 6 pm) to avoid this bias. Research by Moran-Lev et al. has reported that circadian variations in fat concentration appear consistently for the first seven consecutive weeks of lactation, varying by 10.9% between afternoon and morning samples, supporting our observation [22]. Wojcik et al. also showed that the breast milk fat content is positively correlated with afternoon feedings [23]. In the initial phase of breastfeeding, the fat content in human milk may be about 1% (foremilk), and in the final phase, it may increase even up to 9% (hindmilk). However, the degree of breast emptying is deemed to be the strongest influencing factor on the variability of the fat content of HM. In a full breast, this content is lower, and it is higher in an emptied one [24]. In our study, the participants were asked to use an electric pump to drain milk for 7-5-3 min using the full breast, without lactating within the previous 3 h. We observed that, at the start, for most women, breastfeeding came with effort, especially for women with gestational hypertension. We noticed either a lack or a slowly progression of lactogenesis within our study group. Kristin H. et al. drew similar conclusions. The authors reported that women with hypertensive disorders in pregnancy (HDP) are at a higher risk of maternal breastfeeding difficulties, along with a remark on insufficient milk supply [25]. This factor also prevented us from collecting greater volumes of milk to minimize statistical errors.

Depending on the lactation period, the average fat content of breast milk equals 2.6 g/dL in colostrum and up to 4.1 g/dL in mature milk [26]. We analyzed HM samples between the 3rd and 6th day of lactation, within the period of foremilk formation. Our results showed that the mean values of fat, carbohydrate and energy concentrations in the HM of mothers with gestational hypertension were greater in comparison to the control group. Bauer and Gerss evaluated the human breast milk composition of mothers of extremely preterm infants. The authors proved that the carbohydrate and fat concentration of preterm HM increased gradually during the postpartum period of lactation, independently of milk volume [27]. The high fat quantities were responsible for the high energy density, which is concordant with our study results. The fetoplacental units in women with gestational hypertension undergo certain vascular changes. High blood pressure puts pregnant women at risk for emergency cesareans section due to significant blood flow disturbances or placental abruption. We assume that women with gestational hypertension adapt to possible premature birth and, by that means, their mammary glands produce increased amounts of lipids molecules in order to meet the energy needs of a newborn of a lower birth weight, as we also reported in our results.

Obesity in women has a significant impact on pregnancy, and its prevalence continues to raise. In the past decades, the global increase in obesity’s prevalence has been noted, with the number of overweight and obese people tripling between 1975 and 2016 [28]. Accordingly, the incidence of maternal obesity has also been increasing. It does not only affect the mother, but also her offspring, and is associated with various complications, including gestational hypertension, diabetes, preeclampsia, premature delivery and spontaneous abortions, as well as postpartum complications [29,30]. Moreover, we observed that a higher pre-pregnancy BMI predisposes the mother to developing GH. This aspect has been evaluated in other research studies, which have stated that overweight and obesity, as well as excessive weight gain during pregnancy, [24,25] are significant risk factors for GH occurrence [31,32]. This is most likely related to the presence of excessive adipose tissue, which secretes high levels of estrogen. The secretion of aldosterone is also stimulated, inducing the renin-angiotensin system. Endothelial changes at the level of renal tubules and arterioles lead to an increase in blood pressure values [33]. The concentration of individual macronutrients may vary due to numerous maternal factors, including body composition [34]. We have shown no impact of obesity nor overweight on the composition of any macronutrients in the HM of GH women. Specifically, we looked at fat and protein concentration. Our observation is concordant with those of the study by Gabriela L. et al., who found no difference in fat colostrum concentration between overweight, obese and normal-weight women [35]. Another study by Namsoo Ch. et al. showed a declined association between GH and changes in fat concentration in HM [36]. There are suggestions that the HM of obese and overweight mothers’ mature milk may have greater fat concentrations; nevertheless, this has only been observed in mature milk samples so far. A Korean study also showed that the fat concentration increase was most likely due to the higher-fat and/or -protein dietary preferences of obese and overweight women in the postpartum period [37]. In the study by Leghi et al., the authors analyzed various macronutrients’ composition in milk (protein, carbohydrates and fats); yet, it revealed no significant differences between maternal groups according to their body weight statuses. The samples were collected within 2 weeks postpartum [35]. We have not found studies focusing on the association between macronutrient composition in the HM of women with GH in reference to pre-pregnancy BMI to use as a direct referral to our results. Given the considerable variability in the results and questionable quality of the sample collection between various studies, further research is needed to establish the impact of maternal overweight and obesity on HM composition, especially in GH mothers. The association between maternal body mass index and an increased risk of childhood obesity is an important focus of many studies. It has been shown that maternal obesity prior to conception may increase the odds of childhood obesity by up to 264% [38]. Other studies have confirmed this positive correlation [39,40,41]. Therefore, it is essential to acknowledge the need to intervene prior to conception and to manage dietary and physical strategies to support women of childbearing age.

Recent studies have identified gestational hypertension as a significant risk factor for preterm birth [42,43]. Gestational hypertension puts at jeopardy not only the health of the baby, but also of the mother. It is one of the primordial causes of fetal and maternal mortality during the peri-pregnancy period. In our analysis, the majority of births occurred at term, with slight variations between individuals. Only two women with GH gave birth prematurely. Consequently, we were unable to perform a statistical analysis to differentiate the human milk content according to gestational age. Nevertheless, one should be alert and promptly react in cases of high blood pressure values during pregnancy regarding the enduring consequences of prematurity. Furthermore, recently, there have been many studies focused on variance in the composition of breast milk from mothers of preterm babies and newborns at term. It is widely known that the hindmilk from preterm pregnancies contains higher levels of protein, carbohydrates, fats and calories [15]. This may be the response to the more demanding needs of a preterm baby to meet the requirements for its proper development [44]. However, there is no available research focusing specifically on the differences in the content of macroelements in the breast milk of women with gestational hypertension. A more advanced study was performed by Dangat K. et al. The authors investigated the metabolic profiles of milk on the 3rd day and 6th month of lactation within a group of mothers with preeclampsia. They showed an increase in the glycerophosphocholine level (*p* < 0.01) at the 6th month in the human milk of mothers with preeclampsia [17]. They emphasized the need for further investigation of maternal nutrition and characteristics to evaluate its influence on milk composition and investigate the correlation with the infant’s long-term growth. In our research, we plan to evaluate the anthropometric values of infants at the 6th month, from both the study group and the control group, to verify whether there is any correlation between infant growth rate and foremilk composition.

Our study was limited by the small sample size; thus, the results may not be applicable to a greater population, and further studies are needed to confirm our findings. There are numerous factors which may alter milk composition, including maternal diet and vitamin supplementation. Nevertheless, we hope to emphasize the need to broaden the research on gestational hypertension and its long-term maternal and neonatal consequences. The impact of the altered HM composition of GH mothers on the infant growth trajectory will be analyzed and presented after the collection of a greater amount of milk samples. The pre-pregnancy weight statuses were obtained through reviewing medical record. After collection, the milk samples were frozen and then used for analysis. The time of samples’ freezing varied; nevertheless, all of the samples were tested within 3 months after their collection.

Human milk has been the subject of multiple studies, but the influence of pregnancy disorder on its nutritional composition has not yet been well elucidated, and the results of different studies vary significantly. This is why it still remains the target of constant research. We suggest that gestational hypertension may affect the percentage distribution of macronutrients in breast milk, with higher fat and carbohydrate content in the HM of hypertensive women. Differences in the biochemical composition of milk from mothers with hypertension are particularly important when considering their adaptation to the special nutritional needs of newborns from pregnancies of hypertensive mothers, and should be monitored in order to properly fortify mother’s or donor’s milk. This study may help in designing milk formulae for newborns of mothers with gestational hypertension who experience poor lactation or choose not to breastfeed.

## 5. Conclusions

In summary, we found that the human foremilk of women with gestational hypertension had higher fat, carbohydrate and energy concentrations than the human breast milk of normotensive women. Gestational hypertension may play a role in the variation of the macronutrient content of human milk. Moreover, a higher pre-pregnancy BMI may cause a predisposition to gestational hypertension occurrence. Our aim is to further study these correlations, as well as to assess the growth rate of newborns in order to determine the need to formulate individualized formulae for women with pregnancy-induced hypertension with lactogenesis difficulties, or for mothers who cannot or choose not to breastfeed.

## Figures and Tables

**Table 1:** The characteristics of mothers and newborns.

Parameters	Mean ± SD		Median (IQR ^1^)		
**Mothers**	**PIH ^2^**	**NR ^3^**	**PIH ^2^**	**NR ^3^**	***p*-Value**
Age	30.6 ± 4.3	30.1 ± 4.5	30.5 (28–34)	31 (27–33)	0.584
Pre-pregnancy BMI (kg/m^2^)	26.3 ± 5.1	21.5 ± 8.4	24.7 (22.0–30.0)	22.4 (20.3–25.8)	0.005
Pregnancy weight gain (kg)	14.5 ± 6.1	13.1 ± 5.3	15.0 (11.3–19.8)	12.5 (10.0–17.0)	0.323
Pregnancy BMI (kg/m^2^)	31.5 ± 5.1	29.1 ± 4.4	29.7 (27.8–35.1)	28.7 (25.9–31.2)	0.033
**Newborn**	**PIH ^2^**	**NR ^3^**	**PIH ^2^**	**NR ^3^**	***p*-Value**
Birth weight (g)	2951 ± 682	3311 ± 511	3070 (2752–3315)	3320 (3003–3590)	0.013
Body length (cm)	52.1 ± 4.5	54.0 ± 2.4	53.0 (51.0–54.8)	54.0 (52.5–56.0)	0.030
Head circumference (cm)	33.2 ± 2.4	33.8 ± 1.8	33.5 (32–35)	34.0 (33–34.8)	0.312

^1^ IQR, interquartile range; ^2^ PIH, pregnancy-induced hypertension; ^3^ NR, normotensive.

**Table 2:** Correlation between obesity and macronutrient composition of HM in the GH group.

Coefficients				Wald Test		
	Estimate	Standard Error	z	Wald Statistic	*df*	*p*
(Intercept)	−8.986	13.283	−0.677	0.458	1	0.499
Fat	−18.258	15.206	−1.201	1.442	1	0.230
Carbohydrates	−7.482	6.647	−1.126	1.267	1	0.260
Calories	2.100	1.675	1.254	1.573	1	0.210
True protein	−12.766	9.457	−1.350	1.822	1	0.177

**Table 3:** Correlation between overweight and macronutrient composition of HM in the GH group.

Coefficients						
				Wald Test		
	Estimate	Standard Error	z	Wald Statistic	*df*	*p*
(Intercept)	10.225	16.154	0.633	0.401	1	0.527
Fat	−21.476	18.540	−1.158	1.342	1	0.247
Carbohydrates	−10.762	8.618	−1.249	1.559	1	0.212
Calories	2.337	2.025	1.154	1.332	1	0.248
True protein	−13.768	11.588	−1.188	1.412	1	0.235

**Table 4:** The descriptive statistical significance of the impact of gestational hypertension on certain macronutrients’ composition in human breast milk.

Descriptive Statistics								
	Fat		Carbohydrates		Calories		True Protein	
	NR	GH	NR	GH	NR	GH	NR	GH
Valid	38	34	38	34	38	34	38	34
Missing	0	0	0	0	0	0	0	0
Mean	1.9	2.5	7.3	7.7	58.0	63.2	1.9	1.7
Std. Deviation	0.9	0.9	0.5	0.3	8.6	8.0	0.5	0.3
Skewness	0.49	0.34	−1.81	−0.39	0.47	0.01	1.64	1.43
Std. Error of Skewness	0.38	0.40	0.38	0.40	0.38	0.40	0.38	0.40
Kurtosis	−0.57	0.30	4.50	−1.09	−0.10	−0.09	3.70	1.65
Std. Error of Kurtosis	0.75	0.79	0.75	0.79	0.75	0.79	0.75	0.79
Shapiro–Wilk	0.95	0.97	0.85	0.93	0.97	0.97	0.87	0.85
*p*-value of Shapiro–Wilk	0.082	0.623	<0.001	0.027	0.322	0.389	<0.001	<0.001
Minimum	0.6	1.0	5.5	7.1	42.0	49.0	1.2	1.3
Maximum	3.9	4.9	8.0	8.2	77.0	83.0	3.8	2.6

**Table 5:** Comparison of protein content in human breast milk (g/100 mL) in terms of gestational hypertension occurrence.

Independent Samples *t*-Test				
	Test	Statistic	*df*	*p*
True protein	Student	1.470	70.000	0.073
	Welch	1.510	60.958	0.068

Note. For all tests, the alternative hypothesis specifies that group *NO* is greater than group *YES*.

**Table 6:** Comparison of carbohydrate content in human breast milk (g/100 mL) in terms of gestational hypertension occurrence.

Independent Samples *t*-Test				
	Test	Statistic	*df*	*p*
Carbohydrates	Student	−3.532	70.000	<0.001
	Welch	−3.605	65.559	<0.001

Note. For all tests, the alternative hypothesis specifies that group *NO* is less than group *YES.*

**Table 7:** Comparison of fat concentration in human breast milk (g/100 mL) in terms of gestational hypertension occurrence.

Independent Samples *t*-Test				
	Test	Statistic	*df*	*p*
Fat	Student	−2.666	70.000	0.005
	Welch	−2.670	69.434	0.005

Note. For all tests, the alternative hypothesis specifies that group *NO* is less than group *YES.*

**Table 8:** Comparison of energy content in human breast milk (g/100 mL) in terms of gestational hypertension occurrence.

Independent Samples *t*-Test				
	Test	Statistic	*df*	*p*
Energy content	Student	−2.656	70.000	0.005
	Welch	−2.665	69.816	0.005

Note. For all tests, the alternative hypothesis specifies that group *NO* is less than group *YES*.

**Table 9:** Model summary—gestational hypertension.

**Model**	**Deviance**	**AIC**	**BIC**	***df***	**Χ²**	***p***	**McFadden R²**	**Nagelkerke R²**		**Tjur R²**	**Cox and Snell R²**
H_0_	98.300	100.300	102.563	70							
H_1_	70.505	78.505	87.556	67	27.795	<0.001	0.283	0.432		0.325	0.324
**Coefficients**											
							**Wald Test**				
		**Estimate**		**Standard Error**		**z**	**Wald Statistic**	***df***	***p*-Value**		
(Intercept)		−28.379		9.239		−3.072	9.435	1	0.002		
Birth weight		−0.002		0.001		−2.749	7.557	1	0.006		
Carbohydrates		3.779		1.177		3.211	10.311	1	0.001		
3rd trimester BMI		0.158		0.072		2.206	4.868	1	0.027		

Note. Gestational hypertension level ‘*YES*’ coded as class 1.
